# Medical students’ knowledge, attitudes, and practices regarding renal pelvic cancer

**DOI:** 10.1186/s12909-026-09705-7

**Published:** 2026-07-23

**Authors:** Yi Li, Yanfei Feng, Suming Hu

**Affiliations:** 1 Department of Urology, The Second Affiliated Hospital, School of Medicine, Zhejiang University, No. 88 Jiefang Road, Hangzhou, 310009 China; 2 Department of Vascular Surgery, The Second Affiliated Hospital, School of Medicine, Zhejiang University, No. 88 Jiefang Road, Hangzhou, 310009 China

**Keywords:** Renal pelvic cancer, Medical student, Knowledge, Attitude, Practice

## Abstract

**Background:**

This study aimed to investigate the knowledge, attitudes, and practices (KAP) of medical students towards renal pelvic cancer.

**Methods:**

A cross-sectional study was conducted from 17/10/2023 to 15/11/2023, across several universities, gathering a considerable volume of data via a questionnaire survey. Wilcoxon-Mann-Whitney tests or Kruskal-Wallis analysis of variance were performed to compare differences across groups. Spearman correlation analysis was employed to assess the correlations between KAP scores. Multivariable logistic regression analyses were performed to determine the factors influencing KAP.

**Results:**

A total of 907 valid questionnaires were included in the analysis. Among these medical students, 542 (59.8%) were female, while 397 (43.8%) had undertaken an internship or practicum in urological tumors. The median (25th percentile, 75th percentile) score of knowledge, attitude, and practice were 35 (30, 40), 25 (24,28) and 16 (13,20), respectively. Multivariate logistic regression showed that being located in non - Zhejiang province (OR = 2.270, 95% CI: [1.256, 4.105], *P* = 0.007), knowledge score ≥ 35 (OR = 2.609, 95% CI: [1.977, 3.444], *P* < 0.001), and attitude score ≥ 25 (OR = 1.688, 95% CI: [1.272, 2.241], *P* < 0.001) were independently associated with positive practice. The Structural equation modeling (SEM) showed that knowledge directly affected attitude (β = 0.307, *P* < 0.001) and attitude directly affected practice (β = 0.129, *P* = 0.013). In addition, knowledge has both a direct effect on practice (β = 0.404, *P* < 0.001) and another indirect effect on practice through attitude (β = 0.040, *P* = 0.013).

**Conclusions:**

Medical students demonstrate moderate knowledge, positive attitudes, and inactive practices towards renal pelvic cancer. Efforts should be directed towards implementing precise and clinically oriented educational interventions for high-risk and easily overlooked rare diseases such as renal pelvic cancer, with the aim of improving medical students’ abilities in early recognition, risk assessment, referral, and patient management in clinical practice.

**Clinical trial number:**

Not applicable.

**Supplementary Information:**

The online version contains supplementary material available at https://doi.org/10.1186/s12909-026-09705-7.

## Background

Renal pelvic cancer, contributing to 7% of all renal tumors, is classified among the less common types of urological malignancies. It can manifest in any region of the renal pelvis and calyces [1]. Urothelial carcinoma (UC) emerges as the predominant histologic type within renal pelvic cancers [2]. Despite UCs being the fourth most prevalent tumors, upper urinary tract urothelial carcinomas (UTUC), encompassing both renal pelvic and ureteral cancers, are relatively rare. They represent merely 5–10% of all UC cases, with an annual incidence rate in Western countries estimated at almost two cases per 100,000 people [3]. Guidelines from the European Association of Urology (EAU) and findings from numerous clinical studies advocate for a collective treatment approach towards renal pelvic and ureteral cancers, referring to them as UTUC. This unified classification stems from their common mesodermal origin, along with similar etiological factors, clinical manifestations, and diagnostic criteria [4]. The latest data from the American Cancer Society in 2022 indicates that the estimated new incidences of kidney and renal pelvic cancer for both genders are 50,290 for men and 28,710 for women, highlighting the public health importance of these conditions [5]. Besides, the anatomical structure of the renal pelvis and ureter, characterized by thinner muscle layers compared to the bladder, predisposes these areas to rapid tumor invasion through the muscle layer, forming invasive tumors. Consequently, UTUCs often have a poorer clinical prognosis compared to other urothelial malignancies. This reality accentuates the critical need for early prevention, screening, and accurate diagnosis in improving patient outcomes. The most common diagnostic tools include CT scans, ureteroscopy, and urine cytology examinations. However, due to the discomfort and pain associated with ureteroscopy, as well as the requirement for anesthesia, this procedure may not be suitable for all patients [1].

The knowledge, attitude, and practice (KAP) model suggest that an individual’s behavior is significantly influenced by their knowledge and attitudes, a concept that holds considerable importance in understanding health-related behaviors [6, 7]. In public health, this relationship is often explored through KAP surveys to assess knowledge levels, risk perceptions, and behavioral practices. Medical students, as the future physicians tasked with the prevention, diagnosis, and treatment of diseases, including those as challenging as renal pelvic cancer, stand at the forefront of this inquiry. Renal pelvic cancer is characterized by its aggressive nature and poor prognosis, emphasizing the necessity of early prevention, screening, and diagnosis to improve patient outcomes. Although renal pelvic cancer is relatively rare, its clinical significance should not be underestimated. Due to its insidious onset, non-specific early symptoms, and rapid progression, delayed diagnosis and misdiagnosis are common in clinical practice, often resulting in poor prognosis. In particular, insufficient awareness of warning signs such as hematuria and hereditary risk factors including Lynch syndrome may contribute to delayed referral and management. As future frontline clinicians, medical students should therefore possess fundamental competencies in recognizing high-risk presentations and understanding appropriate diagnostic and referral pathways for such malignancies. Given the critical role these future healthcare professionals will play, it is essential they are adequately prepared to implement and advocate for effective health measures. Remarkably, there has been a lack of KAP studies focusing on medical students’ awareness and preparedness regarding relatively rare urological malignancies such as renal pelvic cancer. Existing studies on cancer-related KAP among medical students have primarily concentrated on common malignancies, while evidence regarding less common but high-risk urological tumors remains limited. Furthermore, few studies have explored the underlying relationships among knowledge, attitudes, and practices using structural equation modeling (SEM), leaving an important research gap in understanding the potential mechanisms influencing medical students’ clinical preparedness. In addition, renal pelvic cancer and other uncommon urological malignancies are often underrepresented in undergraduate medical education, resulting in potential educational gaps in early recognition and clinical management. Therefore, this study seeks to address both the research and educational gaps by investigating the knowledge, attitudes, and practices of medical students towards renal pelvic cancer and by further examining the interrelationships among these domains using SEM. The findings may provide evidence to support more targeted and clinically oriented educational strategies for high-risk and easily overlooked diseases in medical training. More specifically, this study aimed to identify educational deficiencies in knowledge, attitudes, and practices regarding renal pelvic cancer among medical students from multiple regions in China and across diverse academic disciplines, including both clinical and non-clinical medical specialties. The study further sought to explore the key factors influencing students’ learning and clinical participation behaviors and to provide empirical evidence for optimizing undergraduate curricula and developing targeted educational interventions in urological oncology. Ultimately, the study seeks to improve medical students’ abilities in the early recognition and clinical management of rare but high-risk urological malignancies.

## Methods

### Study design and participants

This cross-sectional study was conducted between 17/10/2023 and 15/11/2023, 2023, across multiple universities. The sample size was estimated using the standard formula for cross-sectional studies. With a significance level of α = 0.05, a 95% confidence interval (Z = 1.96), an estimated proportion of *p* = 0.5 to obtain the maximum sample size, and a margin of error of δ = 0.05, the minimum required sample size was calculated to be 384 participants. Considering an expected valid response rate of approximately 90%, the minimum target sample size was adjusted to 430 participants. Ultimately, 907 valid questionnaires were included in the final analysis, which substantially exceeded the minimum required sample size and satisfied the statistical requirements for this study. It targeted medical students who were in their third year of study or higher and had undergone internship or practicum experience. Ethical approval for this research was granted by the Ethics Committee of The Second Affiliated Hospital, Zhejiang University School of Medicine [Approval No. 2023(1000)], and informed consent was obtained from all participants. The inclusion criteria specified that participants must be: (1) Students in their third year or higher with hospital internship experience; (2) Willing to participate and able to provide informed consent; (3) Not currently suspended from their studies.

The study utilized convenience sampling to reach students in their third year or beyond from universities within Zhejiang and other provinces. The survey spanned a range of disciplines, including Clinical Medicine, Pharmacy, Preventive Medicine, Traditional Chinese Medicine, Medical Imaging, and Medical Laboratory Science and other specialties. We utilized the Wenjuanxing (Questionnaire Star: https://www.wjx.cn/app/survey.aspx) to generate electronic questionnaires for dissemination. Links to the questionnaire were distributed through WeChat groups consisting of third-year and above students, with the support of university educators and staff from hospital teaching departments. To minimize sampling bias associated with online survey administration, the questionnaire link was distributed across multiple universities and academic disciplines, and participation was voluntary and anonymous. In addition, duplicate submissions were restricted through the questionnaire platform settings, and invalid questionnaires with extremely short or excessively long response times were excluded during data cleaning. The sample size was determined based on methodological criteria using a priori sample size calculation rather than solely on feasibility considerations.

### Questionnaire introduction

Given the limited availability of established KAP instruments specifically tailored to renal pelvic cancer among undergraduate medical students, the questionnaire was newly developed for this study. The construction of the questionnaire was informed by relevant clinical guidelines, educational literature, and previously published KAP studies. Following the initial item generation, the questionnaire underwent systematic content review by two senior urologists from tertiary teaching hospitals who possessed extensive experience in both clinical medical education and urological oncology practice. The experts evaluated item clarity, linguistic ambiguity, content relevance, and appropriateness for undergraduate medical students. Following the expert review, the clinical comprehensiveness of the instrument was systematically evaluated and enhanced. The items within the knowledge dimension were specifically expanded to capture essential cognitive elements concerning early recognition and therapeutic strategies. Two rounds of pilot testing were subsequently conducted among medical students. The first pilot survey included 81 participants and was used to assess item comprehensibility, response patterns, and preliminary questionnaire structure. After revision, a second pilot survey involving 50 participants was conducted to further evaluate the clarity and acceptability of the questionnaire before formal implementation. The final iteration of the pre-test yielded a Cronbach’s alpha coefficient of 0.897 for the overall instrument, indicating high reliability. Specifically, the reliability coefficients were 0.874 for the knowledge section, 0.782 for the attitude section, and 0.906 for the practice section. The suitability of the data structure was evaluated using the Kaiser–Meyer–Olkin (KMO) measure of sampling adequacy. The KMO value obtained in the formal survey was 0.910, indicating excellent sampling adequacy and sufficient inter-item correlations to support the underlying dimensionality of the instrument.

The questionnaire is structured into four primary sections: demographic details, and three evaluative dimensions—knowledge, attitude, and practice. Please refer to the Appendix for a detailed questionnaire. The knowledge section consists of 16 items, with responses ranging from A (very clear − 3 points), B (somewhat clear − 2 points), to C (not clear − 1 point), and a total possible score between 1 and 48 points. The attitude section features 6 items on a five-point Likert scale, from very positive (5 points) to very negative (1 point), with total scores ranging from 6 to 30 points. Similarly, the practice section includes 7 items scored on a five-point Likert scale, from always (5 points) to never (1 point), with scores ranging from 7 to 35 points. Participants scoring above 80% of the total possible points across these dimensions were considered to have adequate knowledge, a positive attitude, and proactive practice behaviors. Those with scores between 60% and 80% were deemed to have moderate levels of knowledge, attitude, and practice. Scores below 60% indicated inadequate knowledge, a negative attitude, and inactive practice [8].

### Statistical analysis

Statistical analysis was conducted using SPSS 26.0 (IBM Corp., Armonk, N.Y., USA). Continuous variables were described using median (25th percentile, 75th percentile), and between-group comparisons were performed using Wilcoxon-Mann-Whitney tests or Kruskal-Wallis analysis of variance. Categorical variables were presented as n (%). Spearman correlation analysis was employed to assess the correlations between knowledge, attitude, and practice scores. In multivariate analysis, the practice score was dichotomized using the median value as the cut-off point, and logistic regression analysis was performed to identify factors associated with relatively higher practice levels. This approach has been commonly adopted in KAP-related studies to facilitate interpretation of factors influencing positive or higher-level practices. In addition, the practice scores in this study were not normally distributed and demonstrated clustering within a relatively limited score range, making dichotomous classification more suitable for identifying characteristics associated with better practice performance. Although dichotomization of continuous variables may reduce statistical power to some extent, the relatively large sample size in this study contributed to the robustness and stability of the regression results. Therefore, this approach was considered appropriate for the exploratory purpose of identifying influencing factors of higher-level practice behaviors. Variables with *P* < 0.10 in univariate analysis were included in the multivariate regression model. This variable selection strategy follows commonly used epidemiological and multivariable modeling principles and has been widely applied in cross-sectional and KAP-related studies to reduce the risk of excluding potentially relevant variables at the preliminary screening stage. Hypotheses were validated using structural equation modeling (SEM) with AMOS software based on the maximum likelihood estimation method. The mediating effects were further examined using the Bootstrap resampling procedure. Bias-corrected 95% confidence intervals (95% CI) were calculated, and mediation effects were considered statistically significant when the confidence interval did not include zero. Model fit was evaluated using multiple fit indices, including the chi-square/degrees of freedom ratio (CMIN/DF), root mean square error of approximation (RMSEA), goodness-of-fit index (GFI), and adjusted goodness-of-fit index (AGFI). If the initial model fit was not ideal, model modification was performed according to modification indices (MI) and clinical-professional rationale. Specifically, covariance paths between residual error terms of semantically or clinically related items within the same latent dimension were released according to the modification indices (MI) to improve model fit. Two-sided *p* < 0.05 were considered statistically significant in this study.

## Results

A total of 1380 questionnaires were distributed, excluding (1) 7 cases of non-respondents; (2) 300 cases with response times less than 90s or greater than 1800s; (3) 42 cases of incomplete or abnormal age information; (4) 123 cases who selected the same option for the entire knowledge section or for both attitude and practice sections, remaining 907 valid questionnaires. The Cronbach’s α coefficient for the formal experiment feedback scale was 0.890 (knowledge dimension α = 0.903, attitude dimension α = 0.741, practice dimension α = 0.841); the KMO value was 0.910.

Of these medical students, 542 (59.8%) were female, with a mean age of 21 (22,22) years, 667 (73.5%) were majoring in clinical medicine, 626 (69.0%) had parents with high school education or below, and 397 (43.8%) had an internship/practicum experience in urological tumors. The median (25th percentile, 75th percentile) score of knowledge, attitude, and practice were 35 (30,40), 25 (24,28) and 16 (13,20), respectively. Medical students who were older than or equal to 22 years old, in their fifth year of college, and majoring in clinical medicine were more likely to have higher knowledge scores. Those with a location out of Zhejiang Province were more likely to have higher practice scores. Those from different schools were more likely to have different knowledge, attitude, and practice scores. Those with an internship/practicum experience in urological tumors were more likely to have higher knowledge and practice scores (*P* < 0.005) (as shown in Table 1).

**Table 1:** Basic information of participants and KAP score

Variable	*N*(%)	Knowledge score		Attitude score		Practice score	
		Median (25th percentile, 75th percentile)	*P*	Median (25th percentile, 75th percentile)	*P*	Median (25th percentile, 75th percentile)	*P*
Total	907	35(30,40)		25(24,28)		16(13,20)	
Age (years)	21(22,22)	/	/	/	/	/	/
Age (years) classification			**< 0.001**		0.336		0.230
< 22	440(48.5)	34(27,39)		26(24,28)		16(13,20)	
≥ 22	467(51.5)	36(32,40)		25(24,28)		17(14,20)	
Gender			0.509		0.453		0.246
Male	365(40.2)	35(30,40)		25(24,28)		17(13,21)	
Female	542(59.8)	35(30,39)		26(24,28)		16(13,20)	
Location [adjusted]			0.126		0.104		**0.010**
Within Zhejiang province	847(93.4)	35(30,40)		25(24,28)		16(13,20)	
Other	60(6.6)	33.5(28,38.5)		24(23,27)		18(14.5,24)	
School [adjusted]			**< 0.001**		**0.013**		**0.025**
Hangzhou Medical College	358(39.5)	36(32,40)		26(24,28)		17(14,20)	
Zhejiang Chinese Medical University	194(21.4)	34(26,39)		25(24,28)		16(12,20)	
Lishui University	96(10.6)	33(28,37)		26(24,28)		15(11,19.5)	
Wenzhou Medical University	54(6.0)	34(29,38)		26(24,27)		17(13,19)	
Zhejiang University	43(4.7)	37(30,42)		24(23,26)		16(12,20)	
Other	162(17.9)	35.5(30,40)		25(24,27)		17(14,21)	
Grade			**< 0.001**		0.653		0.130
Junior	354(39.0)	32(26,37)		26(24,28)		16(12,20)	
Senior	218(24.1)	37(32,41)		25.5(24,28)		17(13,21)	
Fifth year	335(36.9)	36(33,41)		25(24,27)		16(14,20)	
Major [adjusted]			**< 0.001**		0.236		0.118
Clinical Medicine	667(73.5)	36(32,41)		25(24,28)		17(13,20)	
Non-clinical Medicine	240(26.5)	31(25,37)		25(24,27.5)		16(12,20)	
Parents’ education [adjusted]			0.456		0.998		0.168
High school or below	626(69.0)	35(30,39)		25(24,28)		16(13,20)	
College or Bachelor’s Degree and Above	281(31.0)	35(30,40)		25(24,28)		17(13,21)	
Parents are Medical Professionals			0.863		0.190		0.313
Yes	84(9.3)	34(28.5,40.5)		26(24,28)		17(14,20.5)	
No	823(90.7)	35(30,40)		25(24,28)		16(13,20)	
Family or Friends Diagnosed with Urological System Cancer			0.990		0.660		0.354
Yes	42(4.6)	35.5(29,40)		25(23,28)		17(14,20)	
No	865(95.4)	35(30,40)		25(24,28)		16(13,20)	
Internship/Practicum Experience in Urological Tumors			**< 0.001**		0.090		**< 0.001**
Yes	397(43.8)	37(34,42)		26(24,28)		17(14,20)	
No	510(56.2)	33(27,37)		25(24,28)		16(12,20)	

Note: Bold values indicate *P* < 0.05, representing statistical significance

The distribution of knowledge dimension revealed that the question with the highest number of participants choosing the “very clear” option were “Regular check-ups are beneficial for the early detection of renal pelvic cancer, especially in high-risk individuals.” (K4), with 75.1, the question with the highest number of participants choosing the “somewhat clear” option were “Upper urinary tract urothelial carcinoma includes renal pelvic cancer and ureteral cancer.” (K1), with 52.9%, and the question with the highest number of participants choosing the “not clear” option were “Screening for Lynch syndrome is necessary in patients with upper tract urothelial carcinoma (UTUC) and those with a family history of UTUC.” (K7), with 65.2% (as shown in Table 2).

**Table 2:** Knowledge dimension of the participants [n (%) based on the total sample size, *N* = 907]

	A very clear	B somewhat clear	C not clear
K1 Upper urinary tract urothelial carcinoma includes renal pelvic cancer and ureteral cancer.	265(29.2)	**480(52.9)**	162(17.9)
K2 Renal pelvic cancer refers to malignant tumors of the urothelial epithelium mainly originating from the epithelial cells of the renal pelvis or renal calyces.	281(31.0)	**470(51.8)**	156(17.2)
K3 In the majority of cases, early-stage renal pelvic cancer presents with no obvious symptoms, making it easily overlooked.	351(38.7)	**368(40.6)**	188(20.7)
K4 Regular check-ups are beneficial for the early detection of renal pelvic cancer, especially in high-risk individuals.	**681(75.1)**	196(21.6)	30(3.3)
K5 Individuals with a family history of renal pelvic cancer may have a relatively higher risk of developing the disease.	**444(49.0)**	320(35.3)	143(15.8)
K6 Long-term smoking and prolonged exposure to industrial chemicals are risk factors for renal pelvic cancer.	**470(51.8)**	318(35.1)	119(13.1)
K7 Screening for Lynch syndrome is necessary in patients with upper tract urothelial carcinoma (UTUC) and those with a family history of UTUC.	95(10.5)	221(24.4)	**591(65.2)**
K8 The most common symptom of renal pelvic cancer is visible or non-visible hematuria (blood in the urine).	376(41.5)	**388(42.8)**	143(15.8)
K9 In cases of ureteral stones combined with ureteral or renal pelvic cancer, the medical history may be concealed, and symptoms (hematuria) can be masked, leading to potential misdiagnosis.	372(41.0)	**391(43.1)**	144(15.9)
K10 Cystoscopy should be performed when diagnosing renal pelvic cancer to rule out bladder tumors.	364(40.1)	**386(42.6)**	157(17.3)
K11 a: Single lesion, b: Tumor size less than 2 cm, c: High-grade cytology negative, d: Low-grade ureteroscopic biopsy, e: CT shows no invasive features; all these criteria must be met to determine low-risk metastatic renal pelvic cancer.	124(13.7)	**393(43.3)**	390(43.0)
K12 Assessing low-risk cancer patients for nephron-sparing surgery can reduce complications associated with radical surgery while not affecting tumor prognosis, making it the preferred method.	241(26.6)	**439(48.4)**	227(25.0)
K13 After surgery for upper urinary tract urothelial carcinoma, cystoscopy should be performed to exclude bladder cancer recurrence.	280(30.9)	**428(47.2)**	199(21.9)
K14 The treatment of renal pelvic cancer is stage-dependent; localized renal pelvic cancer can be primarily treated with surgery.	279(30.8)	**431(47.5)**	197(21.7)
K15 Recurrent or metastatic renal pelvic cancer requires the use of various treatment methods, including chemotherapy and immunotherapy.	356(39.3)	**417(46.0)**	134(14.8)
K16 Renal pelvic cancer is prone to local progression and metastasis, and advanced or metastatic renal pelvic cancer often has a poorer prognosis.	297(32.7)	**452(49.8)**	158(17.4)

Note: Bold values indicate the response option with the highest percentage for that specific item

Medical students had a relatively positive attitude, with 62.3% strongly believing that early detection of renal pelvic cancer is important to increase the cure rate (A3), and 50.8% strongly believing that enhancing public education and awareness of renal pelvic cancer will help to increase the early diagnosis rate (A5). Meanwhile, 55.7% believed that enhancing knowledge about renal pelvic cancer would be helpful for their future clinical practice (A2) (as shown in Table 3).

**Table 3:** Attitude and practice dimension of the participants [n (%) based on the total sample size, *N* = 907]

Attitude	A very positive	B positive	C neutral	D negative	E completely negative
A1 Do you believe that the knowledge you currently possess will be helpful for your future clinical practice?	227(25.0)	**465(51.3)**	180(19.8)	27(3.0)	8(0.9)
A2 Do you believe that enhancing your knowledge of renal pelvic cancer will be beneficial for your future clinical practice?	293(32.3)	**505(55.7)**	101(11.1)	6(0.7)	2(0.2)
A3 Do you believe in the importance of early detection of renal pelvic cancer for improving cure rate?	**565(62.3)**	313(34.5)	29(3.2)	0(0.0)	0(0.0)
A4 What is your opinion regarding the role of medical students in early screening for renal pelvic cancer?	313(34.5)	**434(47.9)**	136(15.0)	23(2.5)	1(0.1)
A5 Do you believe that strengthening public education and awareness of renal pelvic cancer contributes to an increase in early diagnosis rate?	**461(50.8)**	388(42.8)	51(5.6)	6(0.7)	1(0.1)
A6 Are you willing to participate in screening and prevention campaigns for renal pelvic cancer?	334(36.8)	**444(49.0)**	121(13.3)	8(0.9)	0(0.0)
Practice	A. always	B. often	C. sometimes	D. rarely	E. never
P1 Have you ever been involved in the diagnosis and treatment of patients with renal pelvic cancer during clinical internships or work?	14(1.5)	26(2.9)	114(12.6)	349(38.5)	**404(44.5)**
P2 Do you proactively inquire about information related to the risk of renal pelvic cancer in patients during clinical practice?	23(2.5)	48(5.3)	141(15.5)	**363(40.0)**	332(36.6)
P3 Do you remind patients with renal pelvic cancer to undergo cystoscopy examinations?	74(8.2)	125(13.8)	181(20.0)	258(28.4)	**269(29.7)**
P4 Do you recommend early screening for renal pelvic cancer for high-risk individuals?	202(22.3)	192(21.2)	**214(23.6)**	166(18.3)	133(14.7)
P5 Have you actively enhanced your learning of relevant knowledge about renal pelvic cancer?	31(3.4)	113(12.5)	**341(37.6)**	283(31.2)	139(15.3)
P6 Have you participated in academic activities or research to stay updated on the latest knowledge about renal pelvic cancer?	24(2.6)	47(5.2)	148(16.3)	**349(38.5)**	339(37.4)
P7 Would you engage in educational activities about renal pelvic cancer to raise awareness in society?	91(10.0)	121(13.3)	**289(31.9)**	247(27.2)	159(17.5)

Note: Bold values indicate the response option with the highest percentage for that specific item

Conversely, medical students were less active in their practice, specifically, 37.6% sometimes took the initiative to enhance their knowledge about renal pelvic cancer (P5), 40.0% rarely took the initiative to ask patients for information about the risk of renal pelvic cancer in their clinical practice (P2), and 44.5% were never involved in the the diagnosis and treatment of patients with renal pelvic cancer (P1) (as shown in Table 3).

In the correlation analyses, significant positive correlations were found between knowledge and attitude (*r* = 0.259, *P* = 0.002), knowledge and practice (*r* = 0.367, *P* < 0.001), and attitude and practice (*r* = 0.239, *P* < 0.001), respectively (as shown in Table S1).

Using the median practice score as the cut-off value, multivariate logistic regression showed that being located in non-Zhejiang province (OR = 2.270, 95% CI: [1.256,4.105], *P* = 0.007), knowledge score ≥ 35 (OR = 2.609, 95% CI: [1.977,3.444], *P* < 0.001), and attitude score ≥ 25 (OR = 1.688, 95% CI: [1.272,2.241], *P* < 0.001) were independently associated with relatively higher practice levels (as shown in Table 4).

**Table 4:** Factors of practice based univariable and multivariable logistic regression

Cutoff value: ≥16/<16	No.	Univariable		Multivariable(forward, *P*<0.1)	
		OR(95%CI)	*P*	OR(95%CI)	*P*
Age (years) classification					
< 22	236/440	ref.			
≥ 22	276/467	1.249(0.960,1.625)	0.097		
Gender					
Male	205/365	ref.			
Female	307/542	1.020(0.780,1.332)	0.887		
Location [adjusted]					
Within Zhejiang province	470/847	ref.		ref.	
Other	42/60	1.872(1.060,3.305)	**0.031**	2.270(1.256,4.105)	**0.007**
School [adjusted]					
Hangzhou Medical College	209/358	ref.			
Zhejiang Chinese Medical University	100/194	0.758(0.534,1.078)	0.123		
Lishui University	45/96	0.629(0.400,0.989)	**0.045**		
Wenzhou Medical University	34/54	1.212(0.671,2.188)	0.524		
Zhejiang University	22/43	0.747(0.396,1.408)	0.367		
Other	102/162	1.212(0.827,1.776)	0.324		
Grade					
Junior	191/354	ref.			
Senior	132/218	1.310(0.930,1.845)	0.123		
Fifth year	189/335	1.105(0.818,1.492)	0.516		
Major [adjusted]					
Clinical Medicine	387/667	ref.			
Non-clinical Medicine	125/240	0.786(0.585,1.058)	0.112		
Parents’ education [adjusted]					
High school or below	348/626	ref.			
College or Bachelor’s Degree and Above	164/281	1.120(0.842,1.489)	0.436		
Parents are Medical Professionals					
Yes	53/84	1.356(0.852,2.156)	0.199		
No	459/823	ref.			
Family or Friends Diagnosed with Urological System Cancer					
Yes	27/42	1.410(0.74,2.689)	0.296		
No	485/865	ref.			
Internship/Practicum Experience in Urological Tumors					
Yes	251/397	1.640(1.255,2.144)	**< 0.001**		
No	261/510	ref.			
Knowledge score					
<35	184/425	ref.		ref.	
≥ 35	328/482	2.790(2.127,3.658)	**< 0.001**	2.609(1.977,3.444)	**< 0.001**
Attitude score					
<25	165/354	ref.		ref.	
≥ 25	347/553	1.929(1.472,2.53)	**< 0.001**	1.688(1.272,2.241)	**< 0.001**

Note: Bold values indicate *P* < 0.05, representing statistical significance

**Fig. 1:**
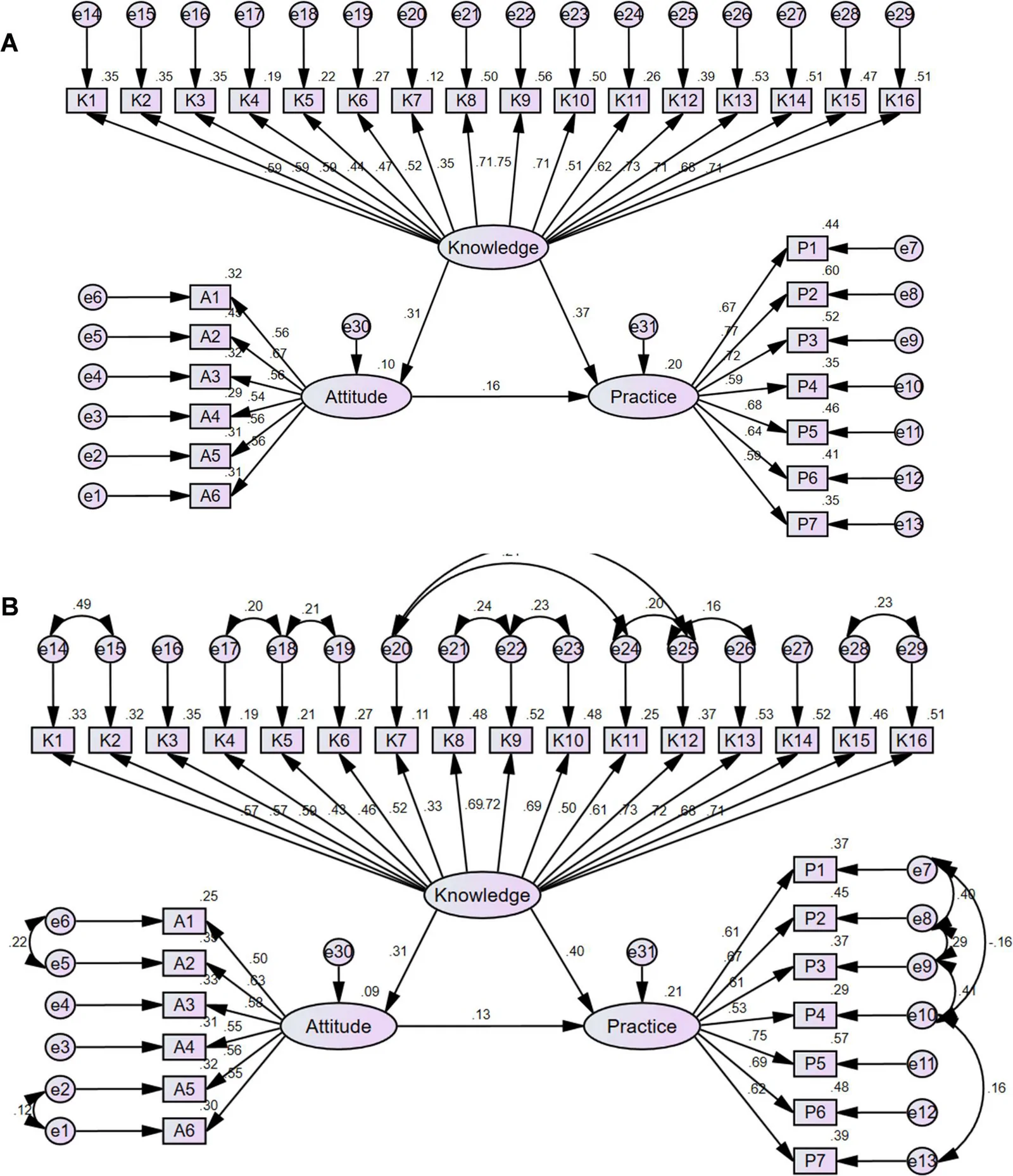
The Structural Equation Model (SEM) Before and After Model Adjustment. **A** Initial model before adjustment; **B** Modified model after adjustment based on modification indices and covariance paths between related residual error terms. Rectangle shows observed variables, ellipses indicate latent variables, and circles represent residual terms

The initial SEM model (Model 1) demonstrated acceptable but not ideal fit indices. Based on the modification indices (MI), correlations between several residual error terms within the same latent dimensions were identified. Specifically, covariance paths were added between residual terms including e14-e15, e17-e18, e18-e19, e20-e25, e21-e22, e24-e25, e25-e26, e28-e29, e5-e6, e1-e2, e7-e8, e8-e9, e9-e10, and e11-e13. These modifications were performed after considering the semantic similarity and professional relevance among related questionnaire items within the same dimensions. After adjustment, the modified SEM model (Model 2) showed improved fit indices, indicating a desirable fit between the model and the observed data. Specifically, the CMIN/DF decreased from 4.216 to 2.387, RMSEA decreased from 0.074 to 0.049, GFI increased from 0.892 to 0.944, and AGFI increased from 0.861 to 0.918 (as shown in Fig. 1). The results showed that knowledge directly affected attitude (β = 0.307, *P* < 0.001) and attitude directly affected practice (β = 0.129, *P* = 0.013). In addition, knowledge has both a direct effect on practice (β = 0.404, *P* < 0.001) and another indirect effect on practice through attitude (β = 0.040, *P* = 0.013) (as shown in Table 5).

**Table 5:** Bootstrap analysis of mediating effect significance test for the final mode

Models	Standardized direct effects	*P*	95%CI		Standardized indirect effects	*P*	95%CI	
			LLCI	ULCI			LLCI	ULCI
K-A	0.307	0.001	0.222	0.394	-	-	-	-
K-P	0.404	0.001	0.326	0.479	-	-	-	-
A-P	0.129	0.013	0.023	0.226	-	-	-	-
K-P	-	-	-	-	0.040	0.013	0.008	0.070

## Discussion

Medical students displayed moderate knowledge, positive attitudes, and inactive practices towards renal pelvic cancer. Given the observed disparities in knowledge, attitude, and practice among medical students, targeted educational interventions focusing on enhancing understanding and fostering proactive engagement with renal pelvic cancer should be implemented within medical curricula to improve patient care and outcomes.

The findings of this study reveal that medical students possess moderate knowledge and positive attitudes towards renal pelvic cancer, yet their practices remain relatively inactive. This situation presents a substantial concern within the realms of medical education and public health efforts. Medical students, throughout their educational journey, encounter a diverse array of medical specialties [9]. Among these, renal pelvic cancer stands out as a comparatively rare urological malignancy [2]. However, despite its relatively low incidence, renal pelvic cancer is closely associated with the broader spectrum of urothelial carcinoma, particularly bladder cancer, which is more commonly encountered in clinical practice. Therefore, the educational significance of renal pelvic cancer extends beyond the disease itself. For medical students, the key learning objectives include recognizing hematuria as an important warning symptom, identifying high-risk populations, understanding appropriate referral pathways, and strengthening awareness of follow-up and surveillance strategies. These competencies are clinically relevant and may help reduce delayed diagnosis and misdiagnosis in patients with urothelial malignancies. Importantly, the practical competencies expected from undergraduate medical students are not equivalent to independently diagnosing or managing renal pelvic cancer, which is typically undertaken in specialized centers. Rather, undergraduate medical education should focus on cultivating fundamental screening, recognition, and referral competencies for rare but highly aggressive malignancies. Renal pelvic cancer is characterized by its aggressive biological behavior, and patient prognosis is highly dependent on timely detection and intervention. In clinical practice, delays in diagnosis frequently occur when early manifestations, such as hematuria, are misattributed to more common conditions including urinary tract infections or urinary calculi, particularly during initial encounters in primary care settings. Consequently, medical students should be trained to recognize warning signs, identify high-risk patients, and initiate appropriate referral pathways when uncommon but potentially serious conditions are suspected. Strengthening these basic practice competencies during undergraduate training may help reduce diagnostic delays, facilitate earlier specialist evaluation, and ultimately contribute to improvements in healthcare quality and long-term patient outcomes. In this context, targeted educational interventions focusing on high-risk and easily overlooked diseases that share common clinical features with more prevalent conditions may provide substantial educational and public health value. The findings also reveal that knowledge and attitudes towards renal pelvic cancer positively correlate with practices, suggesting that enhancing medical students’ understanding and perceptions of this condition may directly influence their practical approaches to diagnosis, treatment, and patient care.

Our study found that age, educational background, and clinical exposure significantly influence knowledge levels and practical application in medical settings. Students aged ≥ 22 years exhibited higher knowledge scores, suggesting that maturity and additional years of education may contribute to a deeper understanding of complex medical conditions like renal pelvic cancer. This observation is consistent with research indicating that older medical students often demonstrate more sophisticated clinical reasoning skills and medical knowledge due to their extended exposure to medical education and training [10]. The significant impact of school affiliation and grade level on knowledge and practice scores indicates that curriculum differences and educational quality across institutions play a critical role in preparing medical students for clinical practice. Notably, students from Hangzhou Medical College and those in higher grade levels showed superior knowledge and practice scores, underscoring the importance of rigorous academic and clinical training programs that emphasize oncology and urology. Furthermore, our study highlights the pivotal role of internship or practicum experience in urological tumors in enhancing both knowledge and practical skills. This supports the notion that hands-on clinical experience is crucial for bridging the gap between theoretical knowledge and practical application in medical practice, a view widely supported in medical education literature [11, 12]. However, the educational implications of this finding extend beyond increasing exposure to a single rare disease. Given the large number of uncommon conditions encountered in clinical medicine, it is neither practical nor necessary for undergraduate curricula to provide extensive disease-specific training for every rare disorder. Instead, specific diseases such as renal pelvic cancer may serve as educational vehicles through which students develop transferable clinical competencies. Through learning about renal pelvic cancer, students can acquire skills that are broadly applicable to other rare but clinically significant conditions, including recognizing atypical presentations, identifying warning signs that warrant further investigation, conducting preliminary risk assessment, understanding appropriate referral pathways, and appreciating the importance of interdisciplinary collaboration. Therefore, educational strategies should not be viewed as a choice between disease-specific exposure and broader competency development. Rather, disease-specific teaching can be used to cultivate generalizable clinical capabilities that support the recognition and management of a wide range of rare diseases throughout future clinical practice. To address the identified gaps, medical schools should consider partnerships with hospitals and research centers specializing in urology to facilitate hands-on learning experiences [13–15]. Moreover, integrating case-based learning and simulation-based training focused on renal pelvic cancer could further enrich students’ learning experiences, ensuring they are better prepared for clinical challenges [16, 17].

The direct and indirect effects of knowledge on practice, mediated by attitudes, underscore the complex interplay between cognitive and affective domains in shaping medical practices. This finding suggests that interventions aimed at improving knowledge and attitudes could have a multiplicative effect on clinical practices, particularly in the early diagnosis and management of renal pelvic cancer. Given these insights, targeted educational interventions that incorporate age-appropriate learning strategies, focus on practical skills development through clinical rotations or internships, and address discrepancies in educational content across institutions could significantly improve outcomes [18, 19]. Additionally, embedding specific modules on renal pelvic cancer, including its early diagnosis, screening, and the importance of hematuria in differential diagnosis, into the medical curriculum could enhance students’ proficiency and practices related to this condition [20, 21].

The knowledge dimension of the participants in this study reveals a varied understanding of renal pelvic cancer, with the highest scoring item being the awareness that regular check-ups are beneficial for early detection, especially in high-risk individuals. This high level of awareness is encouraging and aligns with the literature emphasizing the importance of early detection in improving cancer prognosis [22, 23]. Conversely, the lowest scoring item was the recognition of the necessity for screening for Lynch syndrome in patients with upper tract urothelial carcinoma (UTUC) and those with a family history of UTUC, with 65.2% not clear on this guideline. This gap highlights a critical area for improvement, as understanding genetic risk factors and screening recommendations is essential for comprehensive care in oncology. To enhance knowledge in these areas, it is recommended that medical education curricula incorporate more focused content on genetic aspects of cancers and the role of family history in disease risk assessment [24, 25]. Further, incorporating interactive learning experiences, such as case studies on Lynch syndrome and UTUC, can help solidify students’ understanding of these complex topics. Additionally, guest lectures or seminars by experts in genetic oncology could provide depth to the existing curriculum and inspire interest among students [26, 27].

In the attitude dimension, the strongest positive belief among participants was in the importance of early detection of renal pelvic cancer for improving cure rates, reflecting a commendable recognition of early diagnostic interventions. In contrast, a lower score was observed in the willingness to participate in screening and prevention campaigns, suggesting a potential disconnect between the acknowledged importance of early detection and the willingness to engage in proactive health initiatives. Addressing this discrepancy, educational strategies should aim to not only educate but also motivate students towards active involvement in public health campaigns [28, 29]. Practical recommendations include integrating service-learning components within the medical curriculum, where students can participate in community-based screening and education projects. Such experiences can enhance the applicability of theoretical knowledge, improve attitudes towards public health engagement, and foster a sense of responsibility towards community health [30, 31].

The practice dimension indicates a concerning trend, with a significant portion of students never having been involved in the diagnosis and treatment of patients with renal pelvic cancer. This lack of clinical exposure is a substantial barrier to translating knowledge and positive attitudes into effective practice. Comparatively, a more encouraging aspect is the relatively higher engagement in recommending early screening for high-risk individuals, suggesting that when given the opportunity, students are willing to advocate for preventive measures. It is essential to create more opportunities for hands-on clinical experiences related to renal pelvic cancer. One practical approach could be the incorporation of simulation-based training into the medical curriculum, allowing students to practice diagnostic and treatment procedures in a controlled, educational environment. Additionally, establishing mentorship programs that connect students with oncology specialists for shadowing and clinical rotations could significantly enhance practical skills and reinforce the importance of proactive cancer screening and patient education [32].

Despite its contributions, this study has several limitations. Firstly, the cross-sectional design limits the establishment of causal relationships between knowledge, attitudes, and practices towards renal pelvic cancer among medical students. Secondly, the reliance on self-reported data through questionnaire surveys may introduce response bias and inaccuracies. Third, the study’s focus on medical students from multiple universities within a specific timeframe may limit the generalizability of the findings to broader populations or different contexts. Lastly, this study used convenience sampling and recruited participants through WeChat-based online distribution, which may introduce selection bias. Students with greater interest in oncology-related topics or higher engagement with online learning platforms may have been more likely to participate. Although efforts were made to reduce sampling bias by recruiting participants from multiple universities and disciplines and by applying questionnaire quality control measures, the findings may still not be fully representative of all medical students in different regions or educational settings. Therefore, the generalizability of the findings should be interpreted with caution. However, despite these limitations, the strengths of this paper include its robust sample size, comprehensive assessment of knowledge, attitudes, and practices, and the utilization of advanced statistical analyses such as multivariate logistic regression and structural equation modeling to provide valuable insights into the relationships between these variables.

In conclusion, medical students exhibited moderate levels of knowledge, positive attitudes, and inactive practices concerning renal pelvic cancer. Efforts should be directed towards implementing precise and clinically oriented educational interventions for high-risk and easily overlooked rare diseases such as renal pelvic cancer, with the aim of improving medical students’ abilities in early recognition, risk assessment, referral, and patient management in clinical practice.

## Supplementary Information

Supplementary Material 1.

Supplementary Material 2.

Supplementary Material 3.

Supplementary Material 4.

## Data Availability

All data generated or analysed during this study are included in this published article and its supplementary information files.

## Abbreviations

KAP: Knowledge, attitudes, and practices
UC: Urothelial carcinoma
UTUC: Urothelial carcinomas
