# Supplementary material for: Medical students’ knowledge, attitudes, and practices regarding renal pelvic cancer
Source: BMC Med Educ. 2026 Jul 23;26:1675. doi: 10.1186/s12909-026-09705-7 (PMC13682119; doi:10.1186/s12909-026-09705-7)
Supplement: Supplementary file 1 [file 12909_2026_9705_MOESM2_ESM.docx]

Table S1. Spearman correlation analysis of KAP scores [correlation coefficient (r) and P value]

|  | **Knowledge** | **Attitude** | **Practice** |
| --- | --- | --- | --- |
| **Knowledge** | 1.000 | r = 0.259, P＜0.001 | r = 0.367, P＜0.001 |
| **Attitude** | r = 0.259, P＜0.001 | 1.000 | r = 0.239, P＜0.001 |
| **Practice** | r = 0.367, P＜0.001 | r = 0.239, P＜0.001 | 1.000 |
