# Supplementary material for: Medical students’ knowledge, attitudes, and practices regarding renal pelvic cancer
Source: BMC Med Educ. 2026 Jul 23;26:1675. doi: 10.1186/s12909-026-09705-7 (PMC13682119; doi:10.1186/s12909-026-09705-7)
Supplement: Supplementary file 4 [file 12909_2026_9705_MOESM1_ESM.doc]

Questionnaire number:

Dear friends:

We are researchers from ** Hospital. We sincerely invite you to participate in our research project. This study aims to understand the **medical students' knowledge, attitudes, and practices regarding renal pelvic cancer**, to serve as the basis for developing scientific intervention strategies, which may help many others in the future to improve their health conditions. Your participation in this study is voluntary, and the research has been approved by the Ethics Review Committee. If you agree to participate, please read the following instructions:

1. Please complete the questionnaire. There are no right or wrong answers; you only need to provide responses based on your actual experiences. If you have any questions during the process, feel free to reach out to us, and please submit the completed questionnaire in a timely manner.

2. This study is a simple questionnaire survey and will not cause any harm to your physical or psychological well-being. However, it may involve some personal information such as your gender and age. Please rest assured that we will strictly maintain confidentiality and will not disclose your information.

3. As a participant, you can always stay informed about the information and progress related to this study. If you decide to withdraw from the study, please let us know, and your data will not be included in the research results.

Finally, we sincerely thank you for taking the time to support our scientific research amid your busy schedule!

□I have been informed and agreed to the use of the collected data for scientific research.

Informed Consent Signature：

Date of participation：	Year	Month	Day

| **Part I Basic Information** | |
| --- | --- |
| 1. **Your age： years (integers)** | |
| 2. **Your gender：**a. Male b. Female |  |
| 3. **Province of your school （Province）** |  |
| 4. **Name of your school** |  |
| 5. **Grade：** | A Junior B Senior C Fifth year |
| **6. You are majoring in:** | A Clinical Medicine  B Dentistry  C Public Health and Preventive Medicine  D Integrative Medicine  E Imaging Medicine  F Laboratory Medicine  G Pharmacy |
| **7. Your parents' education level:** | A High school and below  B College or bachelor's degree  C Postgraduate or above |
| **8. Whether any of the parents are medical professionals** | A Yes B No |
| **9. Any family member or friend diagnosed with urological system cancer** | A Yes B No |
| **10. Any internship/practicum experience in urological tumors** | A Yes B No |

| **Part II Knowledge of Renal Pelvic Cancer** | | | |
| --- | --- | --- | --- |
| Depending on your actual situation, please judge how well you understand the following knowledge | | | |
| **1. Upper urinary tract urothelial carcinoma includes renal pelvic cancer and ureteral cancer.** | A.Very clear | B.Somewhat clear | C.Not clear |
| **2. Renal pelvic cancer refers to malignant tumors of the urothelial epithelium mainly originating from the epithelial cells of the renal pelvis or renal calyces.** | A.Very clear | B.Somewhat clear | C.Not clear |
| **3. In the majority of cases, early-stage renal pelvic cancer presents with no obvious symptoms, making it easily overlooked.** | A.Very clear | B.Somewhat clear | C.Not clear |
| **4. Regular check-ups are beneficial for the early detection of renal pelvic cancer, especially in high-risk individuals.** | A.Very clear | B.Somewhat clear | C.Not clear |
| **5. Individuals with a family history of renal pelvic cancer may have a relatively higher risk of developing the disease.** | A.Very clear | B.Somewhat clear | C.Not clear |
| **6. Long-term smoking and prolonged exposure to industrial chemicals are risk factors for renal pelvic cancer.** | A.Very clear | B.Somewhat clear | C.Not clear |
| **7. Screening for Lynch syndrome is necessary in patients with upper tract urothelial carcinoma (UTUC) and those with a family history of UTUC.** | A.Very clear | B.Somewhat clear | C.Not clear |
| **8. The most common symptom of renal pelvic cancer is visible or non-visible hematuria (blood in the urine).** | A.Very clear | B.Somewhat clear | C.Not clear |
| **9. In cases of ureteral stones combined with ureteral or renal pelvic cancer, the medical history may be concealed, and symptoms (hematuria) can be masked, leading to potential misdiagnosis.** | A.Very clear | B.Somewhat clear | C.Not clear |
| **10. Cystoscopy should be performed when diagnosing renal pelvic cancer to rule out bladder tumors.** | A.Very clear | B.Somewhat clear | C.Not clear |
| **11. a: Single lesion, b: Tumor size less than 2 cm, c: High-grade cytology negative, d: Low-grade ureteroscopic biopsy, e: CT shows no invasive features; all these criteria must be met to determine low-risk metastatic renal pelvic cancer.** | A.Very clear | B.Somewhat clear | C.Not clear |
| **12. Assessing low-risk cancer patients for nephron-sparing surgery can reduce complications associated with radical surgery while not affecting tumor prognosis, making it the preferred method.** | A.Very clear | B.Somewhat clear | C.Not clear |
| **13. After surgery for upper urinary tract urothelial carcinoma, cystoscopy should be performed to exclude bladder cancer recurrence.** | A.Very clear | B.Somewhat clear | C.Not clear |
| **14. The treatment of renal pelvic cancer is stage-dependent; localized renal pelvic cancer can be primarily treated with surgery.** | A.Very clear | B.Somewhat clear | C.Not clear |
| **15. Recurrent or metastatic renal pelvic cancer requires the use of various treatment methods, including chemotherapy and immunotherapy.** | A.Very clear | B.Somewhat clear | C.Not clear |
| **16. Renal pelvic cancer is prone to local progression and metastasis, and advanced or metastatic renal pelvic cancer often has a poorer prognosis.** | A.Very clear | B.Somewhat clear | C.Not clear |

| **Part III Attitude to Renal Pelvic Cancer** | | | | | |
| --- | --- | --- | --- | --- | --- |
| **1. Do you believe that the knowledge you currently possess will be helpful for your future clinical practice?** | A.Very convinced | B.Convinced | C.Neutral | D.Not convinced | E.Not convinced at all |
| **2. Do you believe that enhancing your knowledge of renal pelvic cancer will be beneficial for your future clinical practice?** | A.Very convinced | B.Convinced | C.Neutral | D.Not convinced | E.Not convinced at all |
| **3. Do you believe in the importance of early detection of renal pelvic cancer for improving cure rate?** | A.Very convinced | B.Convinced | C.Neutral | D.Not convinced | E.Not convinced at all |
| **4. What is your opinion regarding the role of medical students in early screening for renal pelvic cancer?** | A.Very convinced | B.Convinced | C.Neutral | D.Not convinced | E.Not convinced at all |
| **5. Do you believe that strengthening public education and awareness of renal pelvic cancer contributes to an increase in early diagnosis rate?** | A.Very convinced | B.Convinced | C.Neutral | D.Not convinced | E.Not convinced at all |
| **6. Are you willing to participate in screening and prevention campaigns for renal pelvic cancer?** | A.Very convinced | B.Convinced | C.Neutral | D.Not convinced | E.Not convinced at all |

| **Part IV Practice on Renal Pelvic Cancer** | | | | | |
| --- | --- | --- | --- | --- | --- |
| **1. Have you ever been involved in the diagnosis and treatment of patients with renal pelvic cancer during clinical internships or work?** | A.Always | B.Often | C.Sometimes | D.Rarely | E.Never |
| **2. Do you proactively inquire about information related to the risk of renal pelvic cancer in patients during clinical practice?** | A.Always | B.Often | C.Sometimes | D.Rarely | E.Never |
| **3. Do you remind patients with renal pelvic cancer to undergo cystoscopy examinations?** | A.Always | B.Often | C.Sometimes | D.Rarely | E.Never |
| **4. Do you recommend early screening for renal pelvic cancer for high-risk individuals?** | A.Always | B.Often | C.Sometimes | D.Rarely | E.Never |
| **5. Have you actively enhanced your learning of relevant knowledge about renal pelvic cancer?** | A.Always | B.Often | C.Sometimes | D.Rarely | E.Never |
| **6. Have you participated in academic activities or research to stay updated on the latest knowledge about renal pelvic cancer?** | A.Always | B.Often | C.Sometimes | D.Rarely | E.Never |
| **7. Would you engage in educational activities about renal pelvic cancer to raise awareness in society?** | A.Always | B.Often | C.Sometimes | D.Rarely | E.Never |
